# Diagnosis of Alzheimer’s Disease Based on Accelerated Mirror Descent Optimization and a Three-Dimensional Aggregated Residual Network

**DOI:** 10.3390/s23218708

**Published:** 2023-10-25

**Authors:** Yue Tu, Shukuan Lin, Jianzhong Qiao, Peng Zhang, Kuankuan Hao

**Affiliations:** Department of Computer Science and Engineering, Northeastern University, Shenyang 110819, China

**Keywords:** Alzheimer’s disease, optimization algorithm, mirror descent, CNN, MRI

## Abstract

Alzheimer’s disease (AD), a neuropsychiatric disorder, continually arises in the elderly. To date, no targeted medications have been developed for AD. Early and fast diagnosis of AD plays a pivotal role in identifying potential AD patients, enabling timely medical interventions, and mitigating disease progression. Computer-aided diagnosis (CAD) becomes possible with the burgeoning of deep learning. However, the existing CAD models for processing 3D Alzheimer’s disease images usually have the problems of slow convergence, disappearance of gradient, and falling into local optimum. This makes the training of 3D diagnosis models need a lot of time, and the accuracy is often poor. In this paper, a novel 3D aggregated residual network with accelerated mirror descent optimization is proposed for diagnosing AD. First, a novel unbiased subgradient accelerated mirror descent (SAMD) optimization algorithm is proposed to speed up diagnosis network training. By optimizing the nonlinear projection process, our proposed algorithm can avoid the occurrence of the local optimum in the non-Euclidean distance metric. The most notable aspect is that, to the best of our knowledge, this is the pioneering attempt to optimize the AD diagnosis training process by improving the optimization algorithm. Then, we provide a rigorous proof of the SAMD’s convergence, and the convergence of SAMD is better than any existing gradient descent algorithms. Finally, we use our proposed SAMD algorithm to train our proposed 3D aggregated residual network architecture (ARCNN). We employed the ADNI dataset to train ARCNN diagnostic models separately for the AD vs. NC task and the sMCI vs. pMCI task, followed by testing to evaluate the disease diagnostic outcomes. The results reveal that the accuracy can be improved in diagnosing AD, and the training speed can be accelerated. Our proposed method achieves 95.4% accuracy in AD diagnosis and 79.9% accuracy in MCI diagnosis; the best results contrasted with several state-of-the-art diagnosis methods. In addition, our proposed SAMD algorithm can save about 19% of the convergence time on average in the AD diagnosis model compared with the gradient descent algorithms, which is very momentous in clinic.

## 1. Introduction

**Figure 1:**
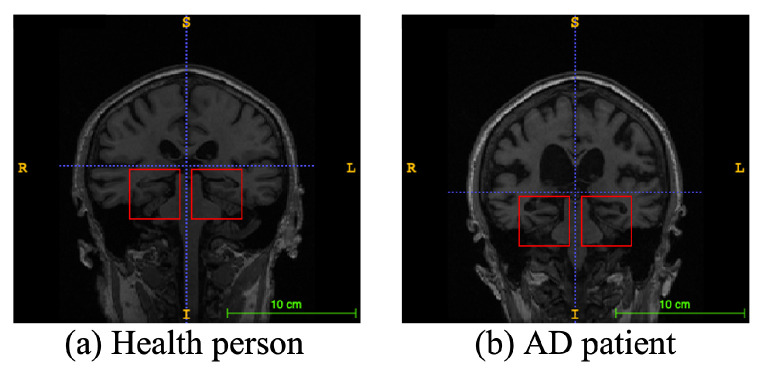
An example of different types of magnetic resonance images. The left image is a healthy person, and the right one is an Alzheimer’s disease patient. The red boxes represent hippocampi.

Alzheimer’s disease (AD), a progressive neurodegenerative disease, is the most typical condition of dementia, mainly in the elderly. It affects people’s memory function, thinking ability, and behavior capability. With the growth of modern life expectancy, the aging phenomenon is aggravating [1]. So, the prevalence of AD is further rising. According to the latest research, patients suffering from AD will reach 74.7 million by 2030 [2], with an average of one person who has AD every three seconds. At present, the etiology of AD is still unclear in medicine, and there is no effective therapeutic method to prevent the incidence and development of AD. Most of the new cases are in low- and middle-income countries. The substantial medical and nursing costs will cause a significant burden to society [3]. Patients with AD usually have organic brain changes, such as narrowing of the gyrus and widening of the sulcus, and especially atrophy of brain tissue, such as the hippocampus and many lobes [4]. Early diagnosis of AD usually used neuroimaging data [5]. Many imaging studies [6] have demonstrated that Alzheimer’s patients have different degrees of hippocampal volume atrophy, the corresponding lateral ventricle enlargement, and temporal angle enlargement phenomena, which gradually extend to the cingulate gyrus and neocortical junction with time. Figure 1 shows an example of different types of magnetic resonance images. In this figure, the left side is the hippocampus of a normal human brain, and the right side is the pathological hippocampus of an AD patient. We can see the atrophy of the hippocampus clearly. These abnormalities, easily obtained by craniocerebral MRI (magnetic resonance imaging), supply essential information for clinicians to diagnose AD.

Moreover, MCI (mild cognitive impairment), a cognitive impairment state between the change in average random cognitive ability and dementia state, has a high probability of eventually becoming AD. MCI is split into two subtypes: sMCI (stable MCI) and pMCI (progress MCI). Patients with sMCI can prevent the development of the disease through drugs and medical intervention. However, patients with pMCI have a high probability of it turning into AD within three years [7]. If AD can be diagnosed early, clinicians can find the probable dementia of patients as soon as possible, carry out drug and medical intervention, and prevent the deterioration of the disease.

The rapid development of neural network technology makes computer-aided AD diagnosis possible. More and more researchers [8,9,10] think about how to use the MRI data of patients for AD diagnosis. The human brain has many functional areas, so the structure of MRI is very complex. When neural networks extract features from complex images, the optimization process is prone to the problems of vanishing gradient [11] and local optimization [12]. Moreover, the craniocerebral MRI data are three-dimensional matrix data. In the process of feature extraction of 3D MRI using neural network models, the training speed of the models is slow, and the accuracy of the diagnosis models is low.

When constructing the 3D MRI CAD model of AD, because data are highly complex, the neural network model often has slow speed in the feature extraction process. At the same time, due to the problems of vanishing gradient [11] and local optimal solutions [12] in the existing optimization algorithms when dealing with complex medical image data, the accuracy of prediction is often not satisfactory. Thus, accelerating the training speed, avoiding the disappearance of partial derivatives in the network training process, and improving accuracy have become challenges of diagnosing AD.

A novel 3D aggregated residual network with subgradient accelerated mirror descent optimization is proposed to tackle the aforementioned challenges. The main contributions are as follows:

• We propose SAMD, an unbiased subgradient accelerated mirror descent optimization algorithm, designed to expedite the training of diagnostic networks. By optimizing and improving the mirror descent algorithm [13], SAMD avoids the optimization problem falling into local optimum under the non-Euclidean distance metric. SAMD achieves accelerated convergence by introducing the step factor and the deviation correction factor to the mirror descent algorithm. Furthermore, the incorporation of a subgradient unbiased estimation mechanism effectively mitigates the issue of vanishing gradient.
• We propose a 3D aggregated residual network (ARCNN) for feature extraction from craniocerebral MRI scans to diagnose AD. The ARCNN leverages our proposed aggregation residual blocks (ARBs) to capture global information within the input data, enhancing its capability to recognize complex patterns and features while improving model stability. Experimental results on the ADNI dataset highlight the superior performance of the diagnostic model trained using the SAMD algorithm in terms of accuracy and efficiency compared to other state-of-the-art methods.
• Our proposed SAMD algorithm achieves a convergence rate that is a square order faster than gradient descent algorithms such as Adam and SGD. Experimental results further validate the efficiency of the SAMD algorithm, showcasing an average time-saving of approximately 19% compared to gradient descent algorithms in the training phase. The SAMD algorithm accelerates AD diagnosis model training, overcoming real-world healthcare computational constraints, ensuring local data security, enabling continuous updates, and enhancing diagnosis efficiency while complying with privacy regulations and evolving medical knowledge.

## 2. Related Work

The vital technology of computer-aided AD diagnosis is to extract neuroimaging features from patients’ MRIs. Generally, the existing AD diagnosis models are separated into four types of feature extraction techniques (i.e., voxel, patch, ROI, and whole-image).

Methods based on voxels [14] attempt to recognize AD-related classification microstructure. Usually, the feature dimension can reach the order of millions, and the number of images used for model training is relatively small (such as dozens). Those make the method face the risk of overfitting.

Comparatively, methods based on the region [15] obtained quantitative features by region segmentation algorithm and then constructed a classifier used to identify patients in a normal control group. Intuitively, these methods only attended to craniocerebral regions defined by experience. Therefore, they may not contain all the possible pathological parts in the cerebrum. Ju et al. [16] used fMRI data to diagnose AD. A brain was divided into 90 regions. According to the Pearson correlation coefficient, the connection of 90 regions is calculated to form a 90×90 relation matrix, which is input to the network for diagnosis.

In order to obtain the changes of local brain regions, a patch-based method [17] used the intermediate scale of feature representation (between the voxel level and region level) to construct a classifier. However, a key question is determining the number, place, and proportions of patches in MR images. Therefore, Lian et al. [10] proposed a CNN (convolutional neural network) to find the voxels with high contrast through statistical analysis and comparison of ordinary images and AD images, and then extracted patches to improve the diagnosis effect.

Wang et al. [18] firstly input 3D MRI into DenseNet and then applied ensemble learning for diagnosis. Cui et al. [19] proposed a diagnostic framework based on CNN and RNN. This method can use the imaging data of different time points. Fang et al. [20] designed a neural network framework, taking advantage of 3D CNN and FSBI-LSTM. It was regarded as a feature extraction to extract the hidden spatial features to promote performance further. Ordinary diagnosis networks used convolutional layers to extract features (such as Resnet [21], Unet [22], etc.), followed by fully connected layers for diagnosis. Because the fully connected layer only processes the one-dimensional information, it resulted in information loss. Fang et al. [20] used bidirectional LSTM instead of fully connected layers to capture spatial information and improve the diagnosis effect. Pan et al. [23] used GAN to generate missing multimodal data for diagnosis. Precisely, the traditional multimodal diagnostic methods discard the subjects without PET (position emission computed tomography) data to solve the problem of modal missing. However, this strategy may significantly reduce training data, thus diminishing the diagnostic performance.

Ji et al. [24] used a deep CNN to diagnose AD, and an effective method was used to train the model using an unbalanced small dataset. Recently, Zhang et al. [25] proposed a diagnostic model with embedding feature selection and feature fusion. They proposed a norm regularization-based optimization algorithm and theoretically demonstrated that it can converge to a global optimum. Their proposed method achieved 84% accuracy in diagnosing AD.

In the existing Alzheimer’s diagnosis models, no matter which feature extraction methods are used for modeling, the optimization algorithms based on gradient descent are used for model training. These training algorithms have some problems, such as local optimization, vanishing gradient, and slow training speed. Therefore, we propose a novel unbiased subgradient accelerated mirror descent optimization algorithm and apply it to our 3D aggregated residual network diagnosis model. The best we can tell is that it is the first time to improve the optimization algorithm of the model in Alzheimer’s diagnosis, which improves the training speed and diagnostic accuracy of the model.

## 3. Materials and Methods

### 3.1. Datasets

The data used in our paper were obtained from the Alzheimer’s Disease Neuroimaging Initiative (ADNI). Parameters of these MR images are listed in Table 1. 348 subjects are separated into 4 categories, including AD, NC (normal control), sMCI, and pMCI. In total, the ADNI dataset contains 78 AD, 100 NC, 117 sMCI, and 53 pMCI subjects.

### 3.2. Proposed Method

**Figure 2:**
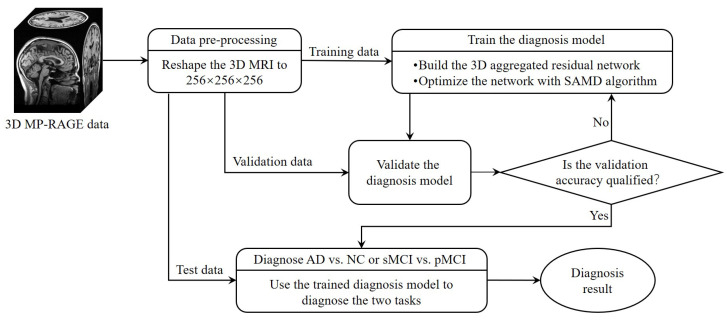
The framework of our proposed diagnostic model.

The whole framework of our proposed diagnostic model is shown in Figure 2. Before training the diagnosis network, data preprocessing (Section 3.2.1) was performed before training. Then, we proposed a 3D aggregated residual diagnosis network (Section 3.2.2) for feature extracting. Our proposed unbiased subgradient accelerated mirror descent optimization algorithm was used in the training process to obtain a faster training speed (Section 3.2.3). Our method addresses two binary classification tasks: diagnosing AD vs. NC and distinguishing sMCI from pMCI. We chose this approach due to clinical relevance; the AD vs. NC task aids early AD diagnosis, while sMCI vs. pMCI classifies cognitive impairment degree. It aligns with disease progression trends, avoiding inconsistencies in a combined model and ensuring meaningful diagnostic insights.

#### 3.2.1. Data Preprocessing

Our dataset comprises numerous subjects, each of which encompasses multiple MR images captured at various time points. We divide the data classified by subjects into MR images one by one and complete all the information of MR images at the same time. In this way, our dataset becomes image-based. The ADNI dataset contains 220 AD, 478 NC, 448 sMCI, and 328 pMCI. By interpolating the original data, every 3D MR image is reshaped into a 256×256×256 data cube, which is suitable as an input of our proposed 3D aggregated residual network.

#### 3.2.2. Three-Dimensional Aggregated Residual Diagnosis Network

**Figure 3:**
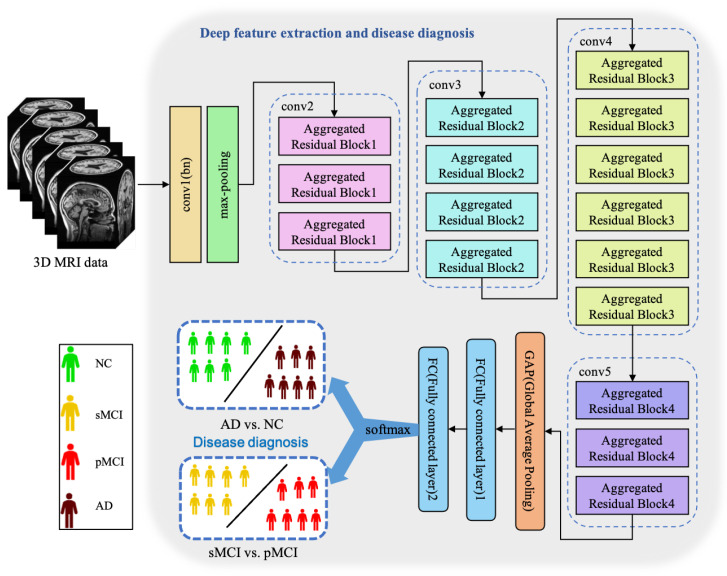
The architecture of our proposed ARCNN. This model achieves two diagnosis tasks so that it needs to be trained respectively.

Essentially, CNN is a feature extractor of layer-by-layer learning mode, which can mine different features from input images. Due to the specific structure of MRI data, specific neural network architectures are required to adequately capture spatial dependencies and features in healthy and ill brains. Because the 3D MRI data of AD patients have large amounts of information, they are often used to deepen or widen the network to enhance the accuracy of feature extraction. The design is conducive to extracting deeper features from MRI, to obtain higher accuracy. However, with the increase in hyperparameters (such as channels, filter size, etc.), the network training cost and design difficulty will greatly increase. We propose a 3D aggregated residual network architecture to extract features from MR images, shown in Figure 3.

**Figure 4:**
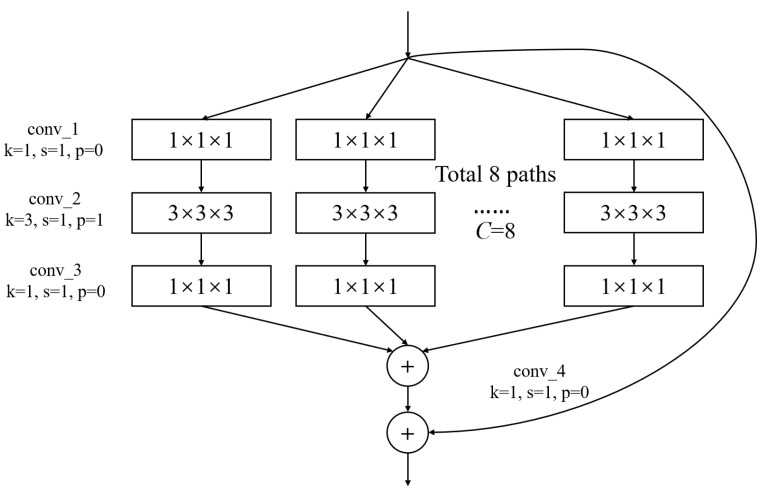
The architecture of ARB. *k* represents the convolutional kernel size, which is a small matrix used for feature extraction in convolutional operations. *p* represents the padding size, which is the technique of adding extra pixels around the input image before convolution to control the output size. *s* represents the stride, which determines the step size at which the kernel moves across the input data.

In Figure 3, conv1(bn) denotes a convolutional layer with batch normalization applied. Conv2-conv5 are aggregated residual blocks (ARBs) with a unique cardinality mechanism to realize the sharing of parameters in the network model, shown in Figure 4. ARBs are designed to aggregate features from multiple preceding layers. This helps the network capture both low-level and high-level information effectively, enabling the model to learn intricate patterns and representations. Using ARB structures can increase the model’s accuracy without significantly increasing the magnitude of parameters. The hyperparameter *C* represents the number of branches in the aggregated feature extraction convolutional layers. In accordance with the data characteristics and experimental conditions, we set *C* to 8. GAP stands for global average pooling, which calculates the average of feature maps, reducing them to scalar values. This reduces data dimensionality, leading to fewer parameters in subsequent fully connected layers and lower computational overhead. As GAP computes the average over all positions, it is spatially invariant, enhancing the model’s ability to handle features at different positions. FC represents the fully connected layer. Table 2 displays details of the diagnostic network structure, including kernels, number of neurons, size of input, and output vectors at each layer. ARCNN is designed to extract neuroimaging features from MRIs, and the network can acquire subtle changes of AD patients’ brain organs to give better diagnosis results.

#### 3.2.3. Accelerated Mirror Descent Optimization

Most diagnostic models of AD are usually 2D, and the extracted brain spatial structure information is insufficient. Therefore, researchers consider using a 3D model for feature extraction. Three-dimensional MRI data contain more features of brain organs, so the training speed of the 3D neural network model is slow. Moreover, training time is still a problem that confines the development of neural network models, and more efficient methods are always sought after. Aiming to accelerate the training speed of the 3D Alzheimer’s disease diagnostic model, a novel accelerated mirror descent algorithm is proposed to decrease training time. This subsection will introduce the proposed unbiased subgradient accelerated mirror descent algorithm (SAMD). Before introducing our proposed algorithm, a table is listed to contain the symbolic representations and definitions used in this subsection, as shown in Table 3.

##### Preliminary

Mathematically, Bregman divergence [26] is defined as a method similar to distance measurement, which extends the square of Euclidean distance to a kind of distance. The distance has no trigonometric inequality and symmetry. Bregman divergence $B_{m}\left(a,b\right)$, with regard to the function m(a), is defined as follows:

(1) $$B_{m}\left(a,b\right)=m\left(a\right)-m\left(b\right)-<\nabla m\left(b\right),a-b>$$

where <a,b> is the scalar product of *a* and *b*. m(·) is a distance generation function.

Assume that m(·) is a strong convex function. Then we have the following inequality [27]:

(2) $$m\left(a\right)\geq m\left(b\right)+<\nabla m\left(b\right),a-b>+\frac{1}{2}\theta_{m}||a-{b||}^{2}$$

where $\theta_{m}$ is a strong convex coefficient.

According to the knowledge of convex optimization, the Bregman divergence $B_{m}\left(a,b\right)$ has the following property:

(3) $$B_{m}\left(a,b\right)\geq \frac{1}{2}\theta_{m}\cdot ||a-{b||}^{2}$$

##### SAMD Optimization Algorithm

The traditional gradient descent algorithm is slow when training data are complex. In the process of optimizing an objective function, the gradient descent algorithm is apt to converge to the local minimum [28]. Moreover, the gradient depends on derivatives, which is imprecise due to the nonsmoothness of the objective function. In addition, numerical schemes need to be used to solve them due to nonsmooth and unknown gradient flows. These gradient flows must have higher-order derivatives than the second-order derivatives. Meanwhile, implementing gradient flow is not easy because numerical artifacts will occur when the derivative is higher than second-order, but the gradient is usually like this.

The optimization objective of the mirror descent algorithm using Bregman divergence as the mirror map is [13]:

(4) $$x_{t+1}={argmin}_{x\in \Omega }\left\{<\nabla F\left(x_{t}\right),x-x_{t}>+L\cdot B_{m}\left(x,x_{t}\right)\right\}$$

where *L* is a Lipschitz constant. *x* indicates the parameters to be trained and Ω is the search space of *x*.

However, for the two items $<\nabla F\left(x_{t}\right),x-x_{t}>$ and $B_{m}\left(x,x_{t}\right)$, there is only one parameter *L* in the projection calculation. In the original mirror descent algorithm (see Equation (4)), constant *L* (called Lipschitz constant) is put forward to regulate the proportion of $<\nabla F\left(x_{t}\right),x-x_{t}>$ and $B_{m}\left(x,x_{t}\right)$. *L* is preset as a hyperparameter when the mirror descent iteration is performed. In the iterative change process, the algorithm cannot dynamically adjust the influence of $<\nabla F\left(x_{t}\right),x-x_{t}>$ and $B_{m}\left(x,x_{t}\right)$ on the iterative results according to the iterative steps. This makes the mirror descent algorithm look a little inflexible. Moreover, the single parameter will make the algorithm oscillate in the iterative process, resulting in the poor effect of the optimization algorithm. So, we use the idea of momentum, which introduces the step factor $\alpha_{t}$ and the deviation correction factor $\beta_{t}$ to dynamically adjust the change speed in the iterative process of the algorithm so that the change of the process tends to be more stable. In this paper, the optimization objective is changed as:

(5) $$x_{t+1}={argmin}_{x\in \Omega }\{\alpha_{t}\cdot <h\left(x_{t}\right),\;x-x_{t}>+\beta_{t}\cdot B_{m}\left(x,\;x_{t}\right)\}$$

Among them, $\alpha_{t}$ is used to control the change of $<\nabla F\left(x_{t}\right),x-x_{t}>$, and $\beta_{t}$ is used to control the change of $B_{m}\left(x,x_{t}\right)$. According to the projection calculation process of the mirror descent algorithm, with iterations, the weights of $\beta_{t}$ and $\alpha_{t}$ change, which can accelerate the speed of the optimization algorithm to the optimal solution to a certain extent. In consideration of the above trends and related knowledge, we set $\alpha_{t}=\frac{1}{t+1}$ and $\beta_{t}=\frac{1}{1-\lambda^{t}}$. $\beta_{t}$ changes with the number of iterations, and the change rate is greater than $\alpha_{t}$.

The mirror descent algorithm is effective when $\nabla F(x_{t})$ can be calculated by a numerical calculation algorithm. However, when the objective function is nonsmooth or the derivative does not exist, $\nabla F(x_{t})$ can not be calculated. At this point, the gradient descent algorithms will not work. Therefore, we use the subgradient instead of traditional gradient in this paper. The subgradient has good performance in solving nondifferentiable convex optimization problems. The subgradients of function F(x) can be regarded as a cluster of supported hyperplanes, represented by ∂F(x). Furthermore, the unbiased estimation of the subgradients E[∂F(x)] is used to replace the gradient so that the results of the numerical analysis are unique, unbiased, and bounded. To prove this point, we give Theorem 1.

**Theorem** **1.** *Given an objective function F(x), for any x∈Ω, there exists a real number N>0 subject to E[∂F(x)]≤N.*

**Proof.**  F(x) is a finite function in a finite set. That is, it is uniformly bounded. Therefore, the unbiased estimation of subgradients is also bounded. The mathematical expectation of a bounded function is bounded. Obviously, ∂F(x) is a bounded function. So, ∀x∈Ω, and there exists a real number N>0 subject to E[∂F(x)]≤N.    □

Based on some knowledge of mathematical analysis, we can prove Theorem 1. Theorem 1 shows that when we calculate the unbiased estimate of the subgradient of F(x), the result is bounded. Hence, E[∂F(x)] can avoid the vanishing gradient problem. E[∂F(x)] can be calculated by the following equation:

(6) $$E\left[\partial F\left(x_{t}\right)\right]=\left\{\begin{array}{c} E\left(\nabla F\left(x_{t}\right)\;\right)\;\;\;\;\;if\;F\left(x\right)\;is\;differentiable\\ E\left(\frac{1}{n}\sum_{j=1}^{n}f_{j}\left(x_{t}\right)\right)\;\;\;\;\;if\;F\left(x\right)\;is\;nondifferentiable \end{array}\right.$$

where $f_{j}\left(x_{t}\right)$ is the sampling of the subgradient hyperplane by F(x) at $x=x_{t}$. By the following equation, the gradient of F(x) can be replaced by h(x)=E[∂F(x)]. This can avoid the vanishing gradient problem.

Finally, the iterative weighted average method is used to control the objective function change further so that the algorithm can achieve the optimal solution faster. We define $\bar{x_{t+1}}$ as the weighted average value in the t+1 iterative step. It can be calculated by the following equation:

(7) $$\bar{x_{t+1}}=\gamma_{t+1}\cdot \bar{x_{t}}+\left(1-\gamma_{t+1}\right)\cdot x_{t+1}$$

where $\bar{x_{t}}$ is the weighted average value in the previous step and $x_{t+1}$ is calculated by Equation (5). $\gamma_{t+1}$ is a dynamically adjusted parameter, which used to adjust the weight between $\bar{x_{t}}$ and $x_{t+1}$ in different iteration steps. $\gamma_{t+1}$ is determined by accumulating the reciprocal of inner product term weight of the current step and the previous step. The accumulation of the reciprocal of inner product term weight at step *t* is represented by $S_{t}$, and the calculation method [29] is:

(8) $$S_{t}=\sum_{i=0}^{t}\frac{1}{\alpha_{t}}$$

This setting can make the iteration step span of each step of the algorithm less large, and can achieve the optimal solution more accurately.

The approach is summarized in Algorithm 1.

Algorithm 1 shows our proposed stochastic unbiased subgradient accelerated mirror descent algorithm. Line 1 gives the initial of the whole algorithm. Lines 3–4 calculate $\alpha_{t}$ and $B_{m}\left(x,x_{t}\right)$, which are put forward to dynamically regulate the impact of $<\nabla F\left(x_{t}\right),x-x_{t}>$ and $B_{m}\left(x,x_{t}\right)$ on the iterative equation (see Line 6). Line 5 calculates our proposed unbiased subgradient method. Lines 7–8 show our proposed iterative weighted average method. By using this method, our proposed SAMD algorithm can converge faster and avoid the error caused by too large a step size. Lines 9–12 are used to evaluate whether the SAMD algorithm fulfills the convergence situation. Through iterations, our proposed SAMD algorithm can train the diagnostic network until it reaches the optimal condition. Then, the convergence of our proposed SAMD will be analyzed below.
**Algorithm 1** A stochastic unbiased subgradient accelerated mirror descent algorithm**Input:** Loss function F(x)**Output:** The optimization result $x^{*}$1: Initial: $t=0,\;x_{0}={argmin}_{x\in \Omega }m\left(x\right)$, obtain λ by grid search2: **while** t≤n **do**3:    Calculate $\alpha_{t}=\frac{1}{t+1}$4:    Calculate $\beta_{t}=\frac{1}{1-\lambda^{t}}$5:    Calculate $h\left(x_{t}\right)=E\left[\partial F\left(x_{t}\right)\right]$6:    Set $x_{t+1}$ by $x_{t+1}={argmin}_{x\in \Omega }\{\alpha_{t}\cdot <h\left(x_{t}\right),\;x-x_{t}>+\beta_{t}\cdot B_{m}\left(x,\;x_{t}\right)\}$7:    Calculate $S_{t}=\sum_{i=0}^{t}\frac{1}{\alpha_{i}}$, $S_{t+1}=\sum_{i=0}^{t+1}\frac{1}{\alpha_{i}}$ and $\gamma_{t+1}=\frac{S_{t}}{S_{t+1}}$8:    Set $\bar{x_{t+1}}$ by $\bar{x_{t+1}}=\gamma_{t+1}\cdot \bar{x_{t}}+\left(1-\gamma_{t+1}\right)\cdot x_{t+1}$9:    **if** $\left|\right|\bar{x_{t+1}}-\bar{x_{t}}\left|\right|<\varepsilon$ **then**10:      Let $x^{*}=\bar{x_{t+1}}$11:      break12:    **end if**13: **end while**14: return $x^{*}$.

##### Convergence of SAMD

The following lemma can be obtained according to the properties of strongly convex functions and Bregman divergence constructed by Bregman function.

**Lemma** **1.** *∀p,q,r∈Ω, we have:*

(9) $$B_{m}\left(p,r\right)-B_{m}\left(q,r\right)=B_{m}\left(p,q\right)+<\nabla m\left(q\right)-\nabla m\left(r\right),p-q>$$

*where constrained set Ω is a closed convex set.*

**Proof.**  According to the definition of Bregman divergence (see in Equation (1)), the following three equations can be deduced:

(10) $$B_{m}\left(p,q\right)=m\left(p\right)-m\left(q\right)-<\nabla m\left(q\right),p-q>$$

(11) $$B_{m}\left(p,r\right)=m\left(p\right)-m\left(r\right)-<\nabla m\left(r\right),p-r>$$

(12) $$B_{m}\left(q,r\right)=m\left(q\right)-m\left(r\right)-<\nabla m\left(r\right),q-r>$$

Then, we have the following process by Equation (11) minus Equation (12):

(13) $$\begin{array}{cc} \; & B_{m}\left(p,r\right)-B_{m}\left(q,r\right)=m\left(p\right)-m\left(q\right)-<\nabla m\left(r\right),p>+<\nabla m\left(r\right),q>\\ \; & \;=m(p)-m(q)-<\nabla m(r),p-q> \end{array}$$

By Equation (13) minus Equation (10), we then have

(14) $$\begin{array}{cc} \; & B_{m}\left(p,r\right)-B_{m}\left(q,r\right)-B_{m}\left(p,q\right)=<\nabla m\left(q\right),p-q>-<\nabla m\left(r\right),p-q>\\ \; & \;=<\nabla m(q)-\nabla m(r),p-q>. \end{array}$$

So, we have $B_{m}\left(p,r\right)-B_{m}\left(q,r\right)=B_{m}\left(p,q\right)+<\nabla m\left(q\right)-\nabla m\left(r\right),p-q>$. □

This lemma is proved by the property of Bregman divergence and is used to obtain the convergence of the SAMD algorithm. Based on some knowledge of mathematical analysis, we can obtain Lemma 2.

To prove the convergence of the unbiased subgradient accelerated mirror descent algorithm, the following lemma can be deduced:

**Lemma** **2.** *When t≥0, for any q∈Ω, we have the follwing inequality:*

(15) $$\begin{array}{cc} \; & \alpha_{t}<h\left(p_{t}\right),p_{t}-q>\leq \beta_{t}\left[B_{m}\left(p_{t},q\right)-E\left[B_{m}\left(p_{t+1},q\right)|\delta_{t}\right]\right]+\frac{{\left(\alpha_{t}N\right)}^{2}}{2\theta_{m}} \end{array}$$

*where $\delta_{t}$ is a sigma-field, i.e., $\delta_{t}=\left\{h\left(p_{0}\right),h\left(p_{1}\right),...,h\left(p_{t}\right)\right\}$, h(p)=E[∂F(p)]. Especially, $\delta_{0}=\delta \left\{p_{0}\right\}$.*

**Proof.**  From the first-order necessary condition, we can obtain

(16) $$<\alpha_{t}h\left(p_{t}\right)+\beta_{t}\left(\nabla m\left(p_{t+1}\right)-\nabla m\left(p_{t}\right)\right),\;q-p_{t+1}>\geq 0$$

Then, it can be acquired that

(17) $$\alpha_{t}<h\left(p_{t}\right),\;p_{t+1}-q>\leq \beta_{t}<\nabla m\left(p_{t+1}\right)-\nabla m\left(p_{t}\right),\;q-p_{t+1}>$$

From Lemma 1, it can be concluded that

(18) $$\begin{array}{cc} \; & <\nabla m\left(p_{t+1}\right)-\nabla m\left(p_{t}\right),\;q-p_{t+1}>=B_{m}\left(p_{t},q\right)-B_{m}\left(p_{t+1},q\right)-B_{m}\left(p_{t},q_{t+1}\right) \end{array}$$

Take Equation (18) into Equation (17), and we can obtain

(19) $$\begin{array}{cc} \; & \alpha_{t}<h\left(p_{t}\right),\;p_{t+1}-q>\leq \beta_{t}\left[B_{m}\left(p_{t},q\right)-B_{m}\left(p_{t+1},q\right)-B_{m}\left(p_{t},p_{t+1}\right)\right] \end{array}$$

Bregman divergence has the following property (see in Equations (2) and (3)):

(20) $$B_{m}\left(p_{t},p_{t+1}\right)\geq \frac{1}{2}\theta_{m}\cdot {∥p_{t}-p_{t+1}∥}^{2}$$

Then, Equation (19) can be transformed to

(21) $$\begin{array}{cc} \; & \alpha_{t}<h\left(p_{t}\right),\;p_{t+1}-q>\leq \beta_{t}\left[B_{m}\left(p_{t},q\right)-B_{m}\left(p_{t+1},q\right)-\frac{1}{2}\theta_{m}{∥p_{t}-p_{t+1}∥}^{2}\right] \end{array}$$

(22) $$\begin{array}{cc} \; & \alpha_{t}<h\left(p_{t}\right),\;p_{t+1}-q>=\alpha_{t}\left(<h\left(p_{t}\right),\;p_{t+1}-p_{t}>+<h\left(p_{t}\right),\;p_{t}-q>\right)\\ \; & \;\geq -\left|<\frac{\alpha_{t}\cdot h\left(p_{t}\right)}{\sqrt{\theta_{m}}},\sqrt{\theta_{m}}\left(p_{t+1}-p_{t}\right)>\right| \end{array}$$

The above inequalities are sorted out:

(23) $$\begin{array}{cc} \; & \alpha_{t}<h\left(p_{t}\right),\;p_{t+1}-q>\geq \alpha_{t}<h\left(p_{t}\right),\;p_{t}-q>-\left|<\frac{\alpha_{t}h\left(p_{t}\right)}{\sqrt{\theta_{m}}},\sqrt{\theta_{m}}\left(p_{t+1}-p_{t}\right)>\right| \end{array}$$

By using the Fenchel inequality, Equation (23) is transformed to

(24) $$\begin{array}{cc} \; & \alpha_{t}<h\left(p_{t}\right),\;p_{t+1}-q>\geq \alpha_{t}<h\left(p_{t}\right),\;p_{t}-q>-\frac{1}{2}\left(\theta_{m}{∥p_{t+1}-p_{t}∥}^{2}+\frac{\alpha_{t}^{2}}{\theta_{m}}{∥h(p_{t})∥}_{*}^{2}\right) \end{array}$$

The above equation is transformed:

(25) $$\begin{array}{cc} \; & \alpha_{t}<h\left(p_{t}\right),\;p_{t}-q>\leq \alpha_{t}<h\left(p_{t}\right),\;p_{t+1}-q>+\frac{\alpha_{t}^{2}}{2\theta_{m}}{∥h(p_{t})∥}_{*}^{2}+\frac{\theta_{m}}{2}{∥p_{t+1}-p_{t}∥}^{2} \end{array}$$

Taking Equation (21) into Equation (25), we have

(26) $$\begin{array}{cc} \; & \alpha_{t}<h\left(p_{t}\right),\;p_{t}-q>\leq \\ \; & \;\beta_{t}\left[B_{m}\left(p_{t},q\right)-B_{m}\left(p_{t+1},q\right)-\frac{\theta_{m}}{2}{∥p_{t+1}-p_{t}∥}^{2}\right]+\frac{\alpha_{t}^{2}}{2\theta_{m}}{∥h(p_{t})∥}_{*}^{2}+\frac{1}{2}{∥p_{t+1}-p_{t}∥}^{2} \end{array}$$

According to Algorithm 1, there has $\beta_{t}=\frac{1}{1-\lambda^{t}}$(λ∈(0,1)), so the value range of $\beta_{t}$ is $\beta_{t}\in \left(1,+\infty \right)$. Then, we take conditional expectations on $\delta_{t}$:

(27) $$\alpha_{t}<h\left(p_{t}\right),p_{t}-q>\leq \beta_{t}\left[B_{m}\left(p_{t},q\right)-E\left[B_{m}\left(p_{t+1},q\right)|\delta_{t}\right]\right]+\frac{{\left(\alpha_{t}N\right)}^{2}}{2\theta_{m}}$$

□

From Lemma 2, we can deduce the following theorem to converge our proposed unbiased subgradient accelerated mirror descent algorithm.

**Theorem** **2.** For an optimization question ${min}_{p\in \Omega }F\left(p\right)$, where F(p) is a strong convex function, $\bar{p_{t}}$ is the optimal solution obtained by the iterative algorithm, and $p^{*}$ is the optimal solution, we have

(28) $$F\left(\bar{p_{t}}\right)-F\left(p^{*}\right)\leq \frac{N^{2}}{2\theta_{m}\left(t+1\right)}$$

**Proof.**  In Lemma 2, let $q=p^{*}$, and we can obtain

(29) $$\begin{array}{cc} \; & \alpha_{t}<h\left(p_{t}\right),\;p_{t}-p^{*}>+\beta_{t}E\left[B_{m}\left(p_{t+1},p^{*}\right)|\delta_{t}\right]\leq \beta_{t}B_{m}\left(p_{t},p^{*}\right)+\frac{\alpha_{t}^{2}N^{2}}{2\theta_{m}} \end{array}$$

By the strong convexity of F(p), we can obtain

(30) $$\begin{array}{cc} \; & \alpha_{t}<h\left(p_{t}\right),\;p_{t}-p^{*}>\geq \alpha_{t}\left[F\left(p_{t}\right)-F\left(p^{*}\right)\right]+\frac{\alpha_{t}\theta_{F}}{2}{∥p_{t}-p^{*}∥}^{2}\geq \alpha_{t}\left[F\left(p_{t}\right)-F\left(p^{*}\right)\right] \end{array}$$

Take Equation (30) into Equation (29). Divide both sides of the inequality by $\alpha_{t}$ and calculate the total expectation:

(31) $$\begin{array}{cc} \; & \frac{\beta_{t}}{\alpha_{t}}E\left[B_{m}\left(p_{t+1},p^{*}\right)\right]+E\left[F\left(p_{t}\right)-F\left(p^{*}\right)\right]\leq \frac{\beta_{t}}{\alpha_{t}}E\left[B_{m}\left(p_{t},p^{*}\right)\right]+\frac{\alpha_{t}N^{2}}{2\theta_{m}} \end{array}$$

Then, we have

(32) $$\begin{array}{cc} \; & E\left[F\left(p_{t}\right)\right]-F\left(p^{*}\right)\leq \frac{N^{2}}{2\theta_{m}\left(t+1\right)}+\frac{\beta_{t}}{\alpha_{t}}\left[B_{m}\left(p_{t},p^{*}\right)-B_{m}\left(p_{t+1},p^{*}\right)\right] \end{array}$$

When the algorithm iterates to $\bar{p_{t}}$, we can come to the following conclusion:

(33) $$F\left(\bar{p_{t}}\right)-F\left(p^{*}\right)\leq \frac{N^{2}}{2\theta_{m}\left(t+1\right)}$$

□

According to Theorem 2, our proposed SAMD algorithm can converge at O(1/t), a square order faster than the gradient descent algorithms (such as Adam and SGD (stochastic gradient descent), which have $O(1/\sqrt{t})$ convergence). It is proved theoretically that the SAMD algorithm proposed in our paper can accelerate the convergence of the training process of the diagnostic network. At the same time, through the relevant description and theorem-proof of the algorithm, it can be obtained that the SAMD algorithm can avoid the problems of vanishing gradient and local optimal solution.

## 4. Experiments and Discussion

We validate our proposed model on the ADNI dataset. In Section 4.1, we describe some settings and performance measures. Section 4.2, Section 4.3, Section 4.4 and Section 4.5 verify the performance of our proposed model.

### 4.1. Experimental Settings

Our proposed model is verified on both AD vs. NC task and sMCI vs. pMCI task. The hardware we utilized consisted of an Intel(R) Core(R) i9-9900K CPU, operating at 3.60 GHz with 16 cores, 16 GB of RAM, a storage setup comprising a 256 GB SSD and a 2 TB SATA drive, along with an NVIDIA RTX 2080 Ti GPU equipped with 11 GB of dedicated memory. This configuration provided the essential computational resources for our experiments.

To assess the performance of our proposed model, we divided the dataset into training, validation, and testing sets. For each class of data, we employed a split ratio of 7:2:1 for these three sets. The training set, comprising 70% of the data, was used for model training, during which the model learned to diagnose diseases by recognizing the features and labels of the data. The validation set, consisting of 20% of the data, played a crucial role in monitoring the model’s performance during training and tuning hyperparameters, aiding in preventing overfitting. The remaining 10% of the data served as the testing set, used to evaluate the model’s ultimate performance. Importantly, the testing set contained data that the model had not previously encountered, ensuring the objectivity of the evaluation. Furthermore, to enhance the evaluation of the model’s performance and generalization capabilities, we ensured that images of the same subject do not appear in more than one of the training, validation, and test sets during the dataset partitioning process.

More specifically, four metrics, namely accuracy (ACC), sensitivity (SEN), specificity (SPE), and area under the curve (AUC, obtained by summing the area under the ROC curve), are used to evaluate our proposed model’s diagnostic performance, which are the most commonly occurring in bioinformatics literature [30,31].

### 4.2. Compared with Other Optimizers

To evaluate the performance of the SAMD optimizer, we train our model and then test on ADNI in the two tasks of diagnosis. The results of SAMD are compared with other mainstream optimization methods by using the same diagnostic networks. Hence, Adam and SGD are chosen as two baselines. Adam, a widely used method in training neural networks, has proved to be better than many gradient methods. SGD is a well-known convex optimization method. Table 4 presents the diagnostic performance of our algorithm in two diagnostic tasks, as compared to other methods, on the testing datasets.

Table 4 shows that the proposed SAMD method has achieved excellent performance in most evaluation indicators. Primarily, our model can diagnose AD with 95.4% accuracy. This better performance is due to the proposed SAMD method being an optimizer based on mirror descent. The proposed method avoids the local optimization and the smoothness limitation of the cost function by constructing the optimization problem in the dual space. Therefore, it can reach the optimal value of the model more accurately. These experimental results also indicate that this model is still effective when the MRI to be diagnosed has different parameters (such as TE, TR) or the distribution of patients is inhomogeneous.

**Figure 5:**
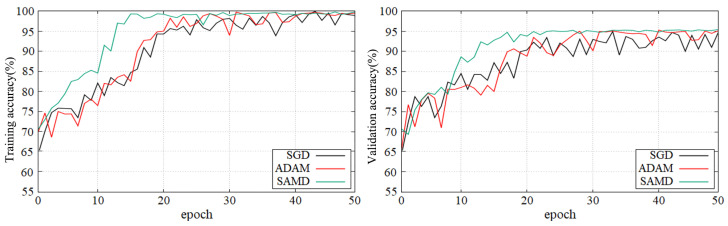
Training and validation accuracy with epochs in AD vs. NC diagnostic task by using ADNI dataset.

**Figure 6:**
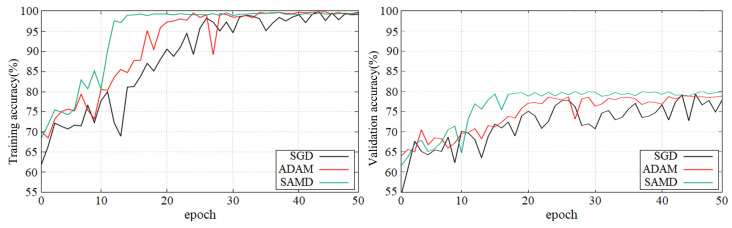
Training and validation accuracy with epochs in sMCI vs. pMCI diagnostic task by using ADNI dataset.

Figure 5 and Figure 6 show the training and validation accuracy of different epochs in two diagnostic tasks. We can obtain that all models are trained to the optimal state from these figures. It is worth noting that the network architecture which used our proposed SAMD optimizer can achieve the optimal state in fewer epochs. That is, our proposed method has a faster convergence speed.

As shown in these two plots, our proposed SAMD method can achieve higher accuracy in a shorter time. Our proposed method, SAMD, can converge faster than other algorithms. Moreover, the model trained by SAMD can achieve higher accuracy eventually. According to the experimental results, we can believe that our proposed SAMD algorithm is effective and efficient in optimizing our proposed AD diagnostic network.

**Figure 7:**
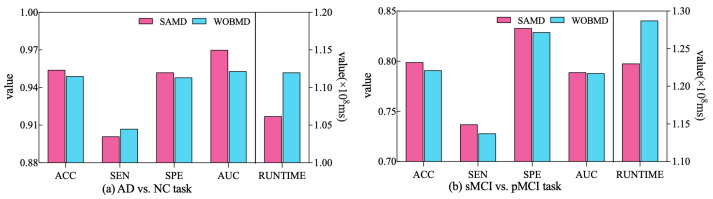
Comparative performance of SAMD and WOBMD.

In order to better demonstrate the efficacy of the proposed adjustment factors in our study, we conducted a comparative experiment. Specifically, we established a control group (referred to as WOBMD) where we set $\beta_{t}$ in SAMD to 1 (i.e., without considering $\beta_{t}$). We applied WOBMD to train and optimize our diagnostic model ARCNN, and subsequently compared the diagnostic outcomes and runtime on both tasks with the results obtained using SAMD, as illustrated in Figure 7. The experimental results reveal that our SAMD algorithm, featuring two adjustment factors, outperforms the WOBMD algorithm, which utilizes only a single adjustment factor. This observation underscores the superiority and innovativeness of our proposed algorithm.

### 4.3. Performance of Diagnostic Network

**Figure 8:**
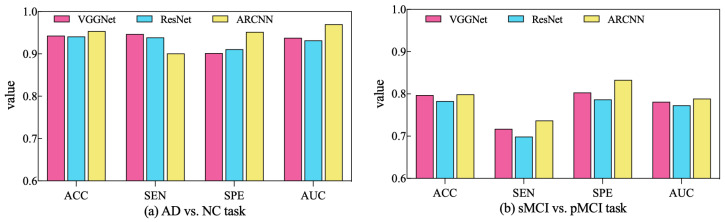
Comparison of diagnostic performance across different diagnostic networks using SAMD as the optimizer in two diagnostic tasks.

In order to assess the performance of the SAMD algorithm when optimizing different diagnostic networks, a series of comparative experiments were conducted. Specifically, two widely employed CNN-based networks in the AD diagnosis field, VGGNet [32] and ResNet [33], were selected for evaluation. SAMD was employed as the optimizer for training and optimizing these two networks on two different diagnostic tasks. The resulting diagnostic outcomes are presented in Figure 8.

As illustrated in Figure 8, SAMD consistently demonstrated good performance across three diagnostic models (including VGGNet, ResNet, and our proposed ARCNN), with particularly superior results observed in the ARCNN model. This observation underscores the adaptability and effectiveness of the SAMD algorithm in optimizing AD diagnostic networks. Furthermore, the experimental results revealed that, in comparison to the two commonly used CNN network architectures, our proposed ARCNN architecture consistently yielded superior diagnostic performance. This demonstrates the advanced and superior characteristics of our model. The experiments confirm the efficacy of the SAMD algorithm in optimizing different diagnostic networks, highlighting its adaptability and effectiveness in the field of medical image analysis, especially for AD diagnosis. Additionally, it is evident that our proposed ARCNN model, with its unique ARB structure, outperforms both ResNet with residual blocks and VGGNet without any special network structure in both diagnostic tasks. This indicates that our proposed ARCNN diagnostic network, featuring ARBs, exhibits advanced performance.

**Figure 9:**
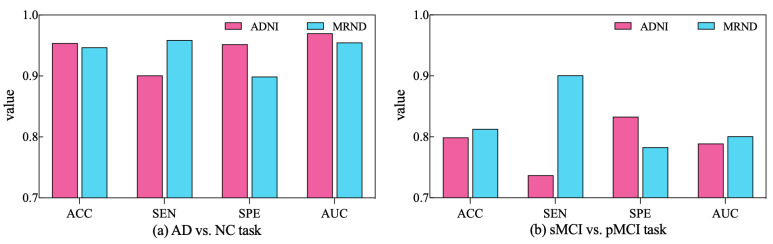
The performance of ARCNN trained with SAMD on MRND.

To further validate the diagnostic performance of our ARCNN model optimized using SAMD, we gathered an additional dataset (represented as MRND), including 67 AD, 89 sMCI, 48 pMCI, and 75 NC. Using the model previously trained on ADNI data, we conducted a performance evaluation with this new MRI dataset, and the results are depicted in Figure 9.

As observed in the figure, our model continues to exhibit superior performance on the new MRI data, indicating the robustness and generalizability of our proposed approach. This outcome underscores the versatility of our method in effectively diagnosing AD across different datasets and highlights its potential for broader clinical applications. The results obtained from the evaluation on the new dataset reaffirm the effectiveness of our SAMD-optimized ARCNN model and its potential for widespread application in AD diagnosis.

### 4.4. Performance of Training Time

**Figure 10:**
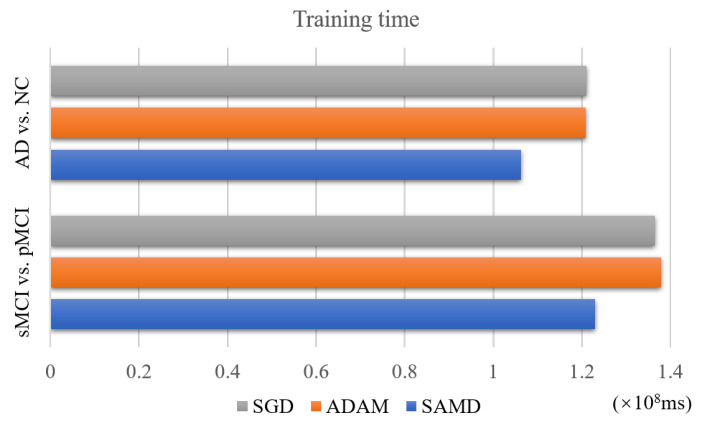
Training time of different optimization algorithms on various tasks.

The SAMD algorithm is proposed to accelerate the training speed of neural networks. The training time of two diagnosis tasks is recorded to verify this point. We applied SAMD, SGD, and Adam optimization to the ARCNN diagnostic model separately and recorded the training times for both diagnostic tasks, as shown in Figure 10. To ensure experimental fairness, we maintained identical datasets and experimental settings across the three different optimization algorithms for ARCNN. In a holistic perspective, the ARCNN model optimized using our proposed SAMD algorithm exhibited an average reduction of approximately 19% in training time compared to models optimized using SGD and Adam when training was completed. The experimental results demonstrate that our proposed SAMD optimization algorithm is capable of accelerating the training process of the ARCNN diagnostic network in different diagnostic tasks.

Furthermore, we present the average inference times for two diagnostic tasks conducted using our proposed diagnostic network. For the AD vs. NC task, the average inference time is 79.5216 ms, while for the sMCI vs. pMCI task, it is 81.3423 ms. It is worth noting that for a well-trained diagnostic model, the process of disease diagnosis is typically instantaneous, requiring minimal waiting time. Consequently, when deploying this system in clinical settings, real-time disease diagnosis can be achieved, facilitating prompt medical interventions and decisions.

### 4.5. Comparison with Previous Work

In Table 5, several SOTA results reported in the literature for AD vs. NC diagnosis and sMCI vs. pMCI diagnosis using structure MRI data of ADNI have been summarized. The underlined values are the optimal values in the baseline methods. From this table, we can see that our proposed method has a more powerful diagnosis capability than the existing machine learning-based AD diagnostic methods. It is worth noting that although many baselines use more data for training than us, our proposed model still achieves the highest accuracy in the two classification tasks. These results demonstrate that our model effectively diagnoses different parametric nuclear magnetic scannings. The 3D aggregated residual diagnosis network has excellent feature extraction ability. The SAMD algorithm can train the neural network model accurately and efficiently, so our proposed architecture can obtain great diagnosis results.

### 4.6. Future Research Directions

In this subsection, we discuss future research directions for AD diagnosis models. Emerging avenues encompass the integration of cutting-edge imaging modalities, such as the fusion of fMRI and PET with MRI images, to bolster early detection capabilities. Furthermore, the development of interpretable AI models is poised to enhance diagnostic transparency, fostering trust within clinical contexts. Additionally, the adoption of early detection strategies, including biomarker discovery and digital health technologies, holds the potential to identify AD in its incipient stages.

The future of AD diagnosis hinges on the amalgamation of advanced imaging, the advancement of interpretable AI models, the realization of early detection, and the prediction of disease progression. Through continuous research endeavors, achieving more efficient and accurate AD diagnosis becomes attainable, enabling timely clinical interventions and treatments, ultimately alleviating societal burdens and enhancing the overall wellbeing of patients.

## 5. Conclusions

Computer-aided diagnosis of AD plays a vital role in helping doctors diagnose disease and find potential patients. We present a novel 3D aggregated residual network with accelerated mirror descent optimization for diagnosing AD. An unbiased subgradient accelerated mirror descent optimization algorithm is proposed to accelerate the training speed of our diagnostic model. The best we can tell is that it is the first work to accelerate the Alzheimer’s diagnosis process by improving the optimization algorithm of the diagnostic model. Theoretically, our proposed SAMD optimization algorithm can converge at an O(1/t) rate. A 3D aggregated residual network is proposed to extract features from 3D MRI data. The experimental results on the ADNI dataset indicate that our proposed method can improve diagnosing AD and achieve 95.4% accuracy in AD diagnosis and 79.9% accuracy in MCI diagnosis. Compared with several SOTA diagnosis models, our proposed model shows excellent performance. In addition, our proposed SAMD algorithm can save about 19% of the convergence time on average compared with several gradient descent algorithms.

We will explore further to improve the disease diagnosis’s efficiency in the future. Moreover, MRI scans are not performed in many hospitals during AD diagnosis. We may consider different kinds of medical images for the diagnosis of AD and make the diagnosis network adaptable for multimodal data.

## Figures and Tables

**Table 1:** Parameters of our acquired dataset.

Parameters	ADNI
Magnetic field intensity	1.5T
Field of view	160
Acquisition matrix	192×192
Thick of scans	1.2 mm
Manufacturer	SIMENS
TE (Echo time)	3.5–3.7 ms
TI (Inversion time)	1000 ms
TR (repetition Time)	3000 ms
Weight	T1

**Table 2:** The topology of our diagnosis network.

Layer	Stage	Input	Size	Output
1	conv1	256 × 256 × 256 × 1	7 × 7 × 7 × 64	128 × 128 × 128 × 64
2	max pool	128 × 128 × 128 × 64	—	64 × 64 × 64 × 64
3	conv2	64 × 64 × 64 × 64	$\left[\begin{array}{c} 1\times 1\times 1\times 4\;\;\;\;\;\;\;\;\;\;\;\;\;\;\;\\ 3\times 3\times 3\times 4,C=8\\ 1\times 1\times 1\times 256\;\;\;\;\;\;\;\;\;\;\;\;\;\; \end{array}\right]\times 3$	64 × 64 × 64 × 256
4	conv3	64 × 64 × 64 × 256	$\left[\begin{array}{c} 1\times 1\times 1\times 8\;\;\;\;\;\;\;\;\;\;\;\;\;\;\;\\ 3\times 3\times 3\times 8,C=8\\ 1\times 1\times 1\times 512\;\;\;\;\;\;\;\;\;\;\;\;\;\; \end{array}\right]\times 4$	32 × 32 × 32 × 512
5	conv4	32 × 32 × 32 × 512	$\left[\begin{array}{c} 1\times 1\times 1\times 16\;\;\;\;\;\;\;\;\;\;\;\;\;\;\;\\ 3\times 3\times 3\times 16,C=8\\ 1\times 1\times 1\times 1024\;\;\;\;\;\;\;\;\;\;\;\; \end{array}\right]\times 6$	16 × 16 × 16 × 1024
6	conv5	16 × 16 × 16 × 1024	$\left[\begin{array}{c} 1\times 1\times 1\times 32\;\;\;\;\;\;\;\;\;\;\;\;\;\;\;\\ 3\times 3\times 3\times 32,C=8\\ 1\times 1\times 1\times 2048\;\;\;\;\;\;\;\;\;\;\;\; \end{array}\right]\times 3$	8 × 8 × 8 × 2048
7	GAP	8 × 8 × 8 × 2048	—	1 × 1 × 1 × 2048
8	FC1	1 × 2048	—	1 × 512
9	FC2	1 × 512	—	1 × 2

**Table 3:** A notation of symbols and definitions.

Name	Meanings
$B_{m}\left(a,b\right)$	Bregman divergence of *a* and *b*
m(·)	Bregman divergence generating function
$\theta_{m}$	Strong convex coefficient
*L*	Lipschitz constant
F(x)	Cost function
<x,y>	Scalar product of *x*, *y*
∇F(x)	Gradient of F(x)
∂F(x)	Subgradient of F(x)
$\alpha_{t}$	Step factor
$\beta_{t}$	Deviation correction factor
$\gamma_{t}$	Dynamically adjusted factor

**Table 4:** Diagnostic performance of two tasks in ADNI dataset.

Methods	AD vs. NC				sMCI vs. pMCI			
	ACC	SEN	SPE	AUC	ACC	SEN	SPE	AUC
SGD	0.947	**0.972**	0.876	0.969	0.779	0.733	0.762	0.787
ADAM	0.951	0.960	0.929	0.967	0.788	0.526	**0.854**	0.781
**SAMD (ours)**	**0.954**	0.901	**0.952**	**0.970**	**0.799**	**0.737**	0.833	**0.789**

Note: The bolded values in each column represent the best-performing values for that specific column.

**Table 5:** Comparison with previous work.

Reference	Subject	AD vs. NC				sMCI vs. pMCI			
		ACC	SEN	SPE	AUC	ACC	SEN	SPE	AUC
Janousova et al. [34]	231 NC + 63 sMCI + 168 pMCI +198 AD	0.880	0.850	0.910	-	0.700	0.640	0.750	-
Liu et al. [35]	77 NC + 85 AD	0.914	0.923	0.904	-	-	-	-	-
Korolev et al. [36]	61 NC + 77 sMCI + 43 pMCI + 50 AD	0.800	-	-	0.870	0.520	-	-	0.520
Karasawa et al. [37]	574 NC + 346 AD	0.940	-	-	-	-	-	-	-
Khvostikov et al. [38]	58 NC + 48 AD	0.854	0.883	0.900	-	-	-	-	-
Lin et al. [39]	229 NC + 188 AD	0.799	0.840	0.748	0.861	-	-	-	-
Xu et al. [30]	165 NC + 95 sMCI +126 pMCI + 142 AD	0.904	**0.924**	0.887	0.954	0.637	**0.786**	0.454	0.679
Cui et al. [31]	223NC + 231 sMCI + 165 pMCI + 192 AD	0.923	0.906	0.937	0.970	0.750	0.733	0.762	0.777
Zhu et al. [40]	419 NC + 345 AD	0.916	0.874	0.948	0.958	-	-	-	-
Poloni et al. [8]	302 NC + 209 AD	0.826	-	-	0.900	-	-	-	-
Alinsaif et al. [9]	50 sMCI +50 pMCI	-	-	-	-	0.700	0.600	0.800	-
Lin et al. [41]	308 NC + 233 sMCI +183 pMCI + 362 AD	0.923	0.904	0.944	0.928	0.741	0.750	0.731	0.766
Gao et al. [42]	427 NC + 342 sMCI +234 pMCI + 352 AD	0.920	0.891	0.940	0.956	0.753	0.773	0.741	0.786
Li et al. [43]	153 NC + 151 AD	0.930	**0.924**	0.948	-	-	-	-	-
**proposed**	100 NC + 117 sMCI + 53 pMCI + 78 AD	**0.954**	0.901	**0.952**	**0.970**	**0.799**	0.737	**0.833**	**0.789**

Note: The bolded values in each column represent the best-performing values for that specific column. The underlined values indicate the best-performing values in the current column among the baselines.

## Data Availability

The dataset used in this study was obtained from Alzheimer’s Disease Neuroimaging Initiative (ADNI). More information regarding ADNI can be obtained from the following link: http://adni.loni.usc.edu/ (accessed on 19 March 2022).
